# Editorial: Efficient artificial intelligence (AI) in ophthalmic imaging

**DOI:** 10.3389/fmed.2024.1523647

**Published:** 2024-12-17

**Authors:** Yanda Meng, Meng Wang, Haoyu Chen, Yalin Zheng

**Affiliations:** ^1^Department of Computer Science, University of Exeter, Exeter, United Kingdom; ^2^Department of Cardiovascular and Metabolic Medicine, University of Liverpool, Liverpool, United Kingdom; ^3^Centre for Innovation and Precision Eye Health, National University of Singapore, Singapore, Singapore; ^4^Joint Shantou International Eye Center, Shantou University and the Chinese University of Hong Kong, Shantou, China; ^5^Department of Eye and Vision Science, University of Liverpool, Liverpool, United Kingdom

**Keywords:** efficient AI, ophthalmic imaging, retinal, ocular, OCT, color fundus

Techniques like optical coherence tomography (OCT), fundus photography, and fluorescein angiography produce a wealth of visual data, offering a detailed view of the eye's structure and function. These imaging tools are essential for identifying and tracking the progression of conditions such as age-related macular degeneration, diabetic retinopathy, and glaucoma. While ophthalmologists are well-trained in reading these images, manually analyzing large datasets can be slow, prone to mistakes, and can vary between observers. Recently, AI tools have shown great promise in supporting ophthalmologists, helping to speed up and improve the accuracy of their diagnoses. This Research Topic comprises nine original research articles covering several different topics using efficient AI, including diabetic macular edema, large language models, macular axial length measurement, ocular surface disease diagnosis, diabetic retinopathy, retinal detachment management, retinal vessel, and microaneurysms segmentation, fundus image registration. A summary of these articles is presented as follows.

Wang et al. introduced an automated framework leveraging deep learning advancements to extract twelve 3D parameters from segmented hyperreflective foci in optical coherence tomography (OCT) images. This development is crucial for understanding various ocular diseases.

Retinal vessels are vital biomarkers for detecting conditions like hypertensive retinopathy. Manual identification is labor-intensive and time-consuming. Liu X. et al. addressed this by proposing a heterogeneous feature cross-attention neural network for retinal vessel segmentation in color fundus images.

Image registration aligns multiple images from different viewpoints or spaces, which is essential in vision applications. Chen et al. introduced an AI-driven approach to unsupervised fundus image registration using a Generalized Polynomial Transformation (GPT) model. Trained on a large synthetic dataset, GPT simulates diverse polynomial transformations.

Microaneurysms, early indicators of diabetic retinopathy, are challenging to detect due to low contrast and similarity to retinal vessels in fluorescein fundus angiography (FFA) images. Li J. et al. presented a model for automatic microaneurysm detection to address these challenges.

Retinal detachment (RD) is a common sight-threatening condition in emergency departments. Early postural intervention based on detachment regions can improve visual prognosis. Li H. et al. developed a weakly supervised model using 24,208 ultra-widefield fundus images to localize and outline anatomical RD regions.

The increasing prevalence of diabetic retinopathy-related (DR-related) diseases among younger individuals poses a significant threat to eye health. Zhao et al. proposed the Neighbored Attention U-Net (NAU-Net) to balance identification performance and computational cost for DR fundus image segmentation.

Pterygium, an ocular surface disease characterized by fibrovascular overgrowth invading the cornea, requires accurate diagnosis. Wan et al. proposed a dual-branch network reinforced by a PFM block (DBPF-Net) for the four-way classification of ocular surface diseases, utilizing a conformer model backbone.

Axial length (AL) is significant for defining the eye's refractive status and is associated with retinal and macular complications. Excessive AL elongation, often over 26.0 mm, increases the risk of posterior segment complications. Liu J. et al. developed deep learning models using macular OCT images to estimate ALs in eyes without maculopathy.

Jin et al. discussed the promising role of large language models (LLMs) in shaping AI's future in ophthalmology. By leveraging AI, ophthalmologists can access information, enhance diagnostic accuracy, and provide better patient care. Despite challenges, ongoing AI advancements and research pave the way for next-generation AI-assisted ophthalmic practices.

